# RES2/406: Making Complex Datasets Available over the Web

**DOI:** 10.2196/jmir.1.suppl1.e78

**Published:** 1999-09-19

**Authors:** N Walker

**Affiliations:** ^1^Institute of Public Health, CambridgeUK

**Keywords:** Anonymity, Confidentiality, Data Dictionary, CGI program, Encryption

## Abstract

**Introduction:**

The internet is the (current) ideal medium for sharing simple data: but the tools for describing complicated datasets, and the ethics and resulting technology for sharing confidential data are less well understood.

**Methods:**

I first describe a simple dataset we've put on the web - some of the world's first genome screen data. The data is anonymous; there was full subject consent; there is no foreseeable subject harm/benefit from data release; and the data sets are in a form readily understood by scientists working in the field. I then describe a large-scale longitudinal epidemiological study, and the tools used to make this comprehensible to secondary data users - the main innovation being a searchable data dictionary and interactive decision support for selecting data subsets from the multi-thousand variable whole. Thirdly I describe the current data access arrangements - "good enough" anonymity, and ftp access for signed-up collaborators. Lastly I describe fully-functioning experimental alternatives: aggregated tables (generated with reference to the data dictionary) and raw data access for named collaborators via encryption, the web's HTTPS protocol using Secure Socket Layers.

**Results:**

Datasets can be shared via the Web, however complex or confidential. For a simple (but important) dataset, see: http://www.mrc-bsu.cam.ac.uk/MSgenetics/ . For a complex dataset and support tools, see: http://www.mrc-bsu.cam.ac.uk/cfas/ or https:// www.mrc-bsu.cam.ac.uk/cfas/. This currently uses US-export (i.e. weak) levels of encryption.

**Discussion:**

There is increasing pressure (from, for example, the Medical Research Council in the UK) to share data collected during publicly-funded medical research. While the social sciences have shared data for many years via archive sites, "patient confidentiality" has prevented it in the medical world. Ironically, the increased use of biological samples- which require far greater stress on confidentiality and the anonymity of public records - have led to proposals for public databases of, and potential competition for, these scarce, expensive resources. For social sciences, record anonymisation is the stripping of identifiers, but they also rely on the fierce legalese of "undertaking forms" to prevent subject identification. This model is breaking down with linked genotypic/phenotypic data - where it might become hugely financially worthwhile to identify a study subject. The data dictionary approach - adopted as an aid to understanding a large complex dataset, can also be used to generate anonymised subsets of the data, and aggregated tables live on the Web. However, full access will require the newer, secure web protocols - if we can find the political and financial will to buy it in from the States.

